# FAM198B promotes colorectal cancer progression by regulating the polarization of tumor-associated macrophages via the SMAD2 signaling pathway

**DOI:** 10.1080/21655979.2022.2075300

**Published:** 2022-05-19

**Authors:** Xiaoxiao Zheng, Jiabin Chen, Tianhao Nan, Li Zheng, Jiahua Lan, Xiaoqin Jin, Ying Cai, Hao Liu, Wei Chen

**Affiliations:** a College of Life Sciences, Zhejiang Chinese Medical University, Hangzhou, Zhejiang, China; b Cancer Institute of Integrated Traditional Chinese and Western Medicine, Key Laboratory of Cancer Prevention and Therapy Combining Traditional Chinese and Western Medicine, Zhejiang Academy of Traditional Chinese Medicine, Hangzhou, Zhejiang, China; c Academy of Chinese Medical Sciences, Zhejiang Chinese Medical University, Hangzhou, Zhejiang, China

**Keywords:** Colorectal cancer (CRC), tumor-associated macrophages (TAMs), FAM198B, SAMD2, macrophage polarization

## Abstract

Colorectal cancer (CRC) is one of the most common malignant tumors. Tumor-associated macrophages (TAMs) promote the progression of CRC, but the mechanism is not completely clear. The present study aimed to reveal the expression and function of FAM198B in TAMs, and the role of FAM198B in mediating macrophage polarization in CRC. The role of FAM198B in macrophage activity, cell cycle, and angiogenesis was evaluated by CCK-8 assay, flow cytometry, and vasculogenic mimicry assay. The effects of FAM198B on macrophage polarization were determined by flow cytometry. The function of FAM198B-mediated macrophage polarization on CRC progression was evaluated by transwell assays. Bioinformatic analyses and rescue assays were performed to identify biological functions and signaling pathways involved in FAM198B regulation of macrophage polarization. Increased FAM198B expression in TAMs is negatively associated with poor CRC prognosis. Functional assays showed that FAM198B promotes M2 macrophage polarization, which leads to CRC cell proliferation, migration, and invasion. Mechanistically, FAM198B regulates the M2 polarization of macrophages by targeting SMAD2, identifying the SMAD2 pathway as a mechanism by which FAM198B promotes CRC progression through regulating macrophage polarization. These findings provide a possible molecular mechanism for FAM198B in TAMs in CRC and suggest that FAM198B may be a novel therapeutic target in CRC.

## Graphical abstract

**Figure:**
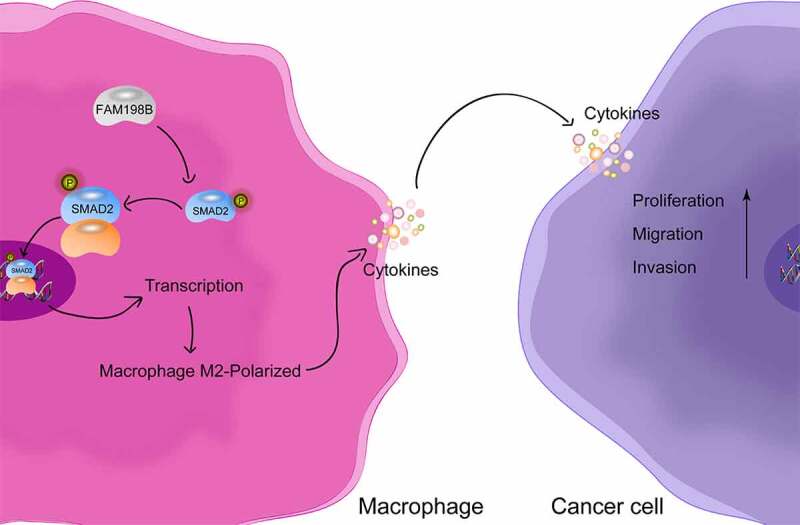

## Highlights

- Increased FAM198B expression of macrophages in CRC is associated with poor prognosis.
- The function of FAM198B in macrophages.
- FAM198B promotes M2 macrophages polarization.
- High expression of FAM198B in macrophages promotes the progression of CRC in vitro.
- Protein-protein Interaction Enrichment Analysis of FAM198B.
- Genes that may be regulated by FAM198B in macrophage polarization.
- FAM198B regulates the M2 polarization of macrophages by targeting SMAD2.

## Introduction

Colorectal Cancer (CRC) is a lethal cancer with an increasing incidence worldwide [1]. Despite advances in the treatment of CRC over the past few decades, the mortality of CRC remains high, mainly due to recurrence and distant organ metastasis [2]. Therefore, uncovering the molecular mechanisms of CRC metastasis and determining new molecular targets are crucial in the development of novel and more effective treatments for this deadly malignancy. The tumor microenvironment (TME) is complex, and includes tumor cells, tumor-associated macrophages (TAMs), stromal cells, and acellular constituents. TAMs are a major component of the tumor microenvironment [3] and in the context of cancer are widely polarized into a M2-like state, which is selectively activated. M2 macrophages play important roles in tumor-promoting functions, including tumor metastasis, angiogenesis, growth, immunosuppression, and therapeutic resistance [4,5]. Therefore, targeting tumor-associated macrophages as a may be a potential novel therapeutic strategy in the treatment of CRC.

FAM198B (family with sequence similarity 198 member B) is a poorly described protein. The effects of FAM198B are rarely discussed, and FAM198B is predicted to be a membrane-bound glycoprotein localized to the Golgi apparatus [6,7] and may be a gene that is related to PAC resistance [8]. While it has been reported that the CELF2/FAM198B axis regulates proliferation and metastasis in ovarian cancer [9], FAM198B was also shown to prolong survival and inhibit metastasis in lung adenocarcinoma by blocking ERK-mediated MMP-1 expression [10]. Our team has been studying the roles of tumor-associated macrophages for a long time [11], and the objective of our current study is to reveal the role and potential mechanism of action of FAM198B in colorectal cancer tumor-associated macrophages.

Smad2 is a signal transducer and transcription factor and plays an important role in the canonical transforming growth factor-β (TGF-β) signaling pathway [12,13]. TGF-β1 can inhibit or change the activation, maturation, and differentiation of a variety of cells, such as macrophages, dendritic cells (DCs), and neutrophils [14]. Smad2/TGF-β1 signaling regulates the M2 polarization of macrophages, which is one of the key events in pulmonary fibrosis [15]. Smad2/TGF-β1 strictly regulates the function of macrophages by mediating the phenotype of phagocytes and promoting anti-inflammatory transformation in infarcted heart tissue [16]. SMAD2/TGF-β1 signaling also has an important role in cancer, including in CRC [17]. While the effects of Smad2 on macrophage function in CRC are unclear, this study may clarify whether there is a relationship between SMAD2 and macrophage polarization in CRC.

In this study, we demonstrate that FAM198B induces macrophage M2 polarization and has important effects on the cell cycle, cell viability, and angiogenesis of macrophages. Using a co-culture system of macrophages and CRC cells, we show that FAM198B induces CRC metastasis through regulating macrophage polarization; SMAD2 knockdown reversed these effects. These data indicate that FAM198B promotes the metastasis of CRC through the SMAD2 signaling pathway. These data indicate that targeting FAM198B in macrophages may be an effective strategy to inhibit CRC metastasis.

## Methods and materials

### Data from public databases: TCIA (TCGA), TISCH, GEPIA

The Cancer Immunome Database (TCIA) internet site: https://www.tcia.at[18]

Tumor Immune Single-cell Hub (TISCH) internet site: http://tisch.comp-genomics.org/home[19] GEPIA internet site:http://gepia.cancer-pku.cn[20]

### Cell lines and materials

The THP-1 and LoVo cell lines were purchased from the Procell Life Science & Technology Co., Ltd. Cell lines were maintained in RPMI-1640 or Ham’s F-12 K supplemented with 10 mM HEPES, 0.05 mM β-mercaptoethanol, 100 U/mL penicillin, 100 g/mL streptomycin (all from Gibco), and 10% fetal calf serum (Gibco) at 37°C in a 5% CO_2_ atmosphere.

### siRNA transfection

THP-1 cells were transfected with FAM198B siRNA (sc-88915, 10 mM, Santa Cruz, California, USA) or negative control siRNA using Opti-MEM (Invitrogen, Carlsbad, USA) and Lipofectamine 2000 (Invitrogen), according to the manufacturers’ instructions. Transfection medium was replaced with a complete medium 6 h after transfection. All subsequent experiments were performed 24 h after transfection and were repeated three times.

### Flow cytometry

THP-1 cells were washed with PBS, centrifuged, and immersed in cell cycle staining reagent (Dojindo). After 30 min incubation at 4°C in the dark, cells were washed with PBS analyzed by flow cytometry (BD). For cell cycle studies, THP-1 cells were washed with PBS, centrifuged, and resuspended in a working solution (Cell Cycle Assay Kit-PI/RNase Staining; Dojindo). The cells were incubated at 4°C for 30 minutes in the dark and then analyzed using a FACSort flow cytometer (BD, San Jose, CA). The ModFit LT program version 2.0 (Verity Software, Topsham, ME) was used to determine the percentage of cells in each stage of the cell cycle. For the surface protein expression of CD16/32, CD206, and CD163, the cells were labeled with phycoerythrin-labeled anti-human CD16/32, CD206, or CD163 antibodies (Biolegend, CA, USA) in a phosphate solution and incubated at 4°C for 30 minutes in the dark, then incubated with a buffered saline (PBS; GIBCO, CA, USA) containing 2% bovine serum albumin (BSA; Sigma, CA, USA) in PBS for 30 minutes. Dead cells were excluded based on propidium iodide staining.

### CCK-8 cell viability assay

The Cell Counting Kit 8 (CCK-8) is used to evaluate cell viability. THP-1 cells transfected with FAM198B siRNA or NC siRNA (5 × 10^3^ cells/well) were inoculated into 96-well plates and were cultured in medium containing 1% serum to synchronize cells for 24 h. CCK-8 working solution (10 ul/well, 100 ul common medium) was incubated for different time points, and the absorption peak was detected by a microplate reader at 450 nm to calculate the relative cell viability.

### Vasculogenic mimicry assay

The effects of FAM198B on angiogenesis were investigated by a tubule formation assay. Briefly, Matrigel (BD Biosciences) was added to a precooled 24-well plate (100 ul Matrigel per well) and allowed to set at 37° for 30 min. HUVEC cells (1 × 10^5^ cells/well) were inoculated into 24-well plates and cultured with cell supernatants of different treatments for 6 hours, then the tubular structures were photographed. Each measurement condition was evaluated from three separate experiments. Images were taken with an Olympus microscope (Olympus).

### Protein isolation and immunoblot analysis

THP-1 cells were suspended in a 100 μL cell lysis buffer containing a phosphatase and protease inhibitor cocktail (Sigma). Protein concentration was measured by BCA protein assay (Thermo) and then analyzed by sodium dodecyl sulfate-polyacrylamide gel electrophoresis (SDS-PAGE). An equivalent amount of sample (20 µg protein) was loaded in each well. The protein lysate was then subjected to SDS-PAGE and transferred to Immobilon-P PVDF membrane (PVDF). Blots were incubated overnight in primary antibodies at 4°C. The antibodies used were anti-FAM198B (Proteintech), anti-STAT6, anti-STAT3, anti-STAT2, anti-STAT1, anti-Smad2, anti-P-smad2 (Ser465/467), and anti-P-smad2 (Ser245/250/255), purchased from Cell Signaling Technology (CST). On the following day, blots were incubated with the appropriate HRP-coupled secondary antibodies at 4°C for 2 hours. Chemiluminescence was performed using the ECL detection kit (Bioworlde). Band density was estimated, and protein levels were normalized to GAPDH.

### In vitro cell migration, invasion and clone assay

The effects of macrophage FAM198B expression on CRC migration and invasion were explored by using a co-culture system. CRC cells were inoculated in the upper chamber of a Transwell 24 insertion plate with a medium at a density of 2 × 10^5^ cells/well. The upper chamber of the wells were coated (invasion assay) with Matrigel (BD Biosciences) or were uncoated (migration assay), and THP-1 FAM198B knockdown cells or FAM198B overexpressing cells were seeded in the lower chamber containing culture medium with 10% serum. After 8–24 hours of culture, the upper chamber was fixed with methanol for 10 minutes and stained with crystal violet. The unmigrated/noninvaded cells were carefully wiped off. The migrated and invaded cells were quantified or counted under an inverted phase contrast microscope (Olympus) and photographed. THP-1 FAM198B overexpression or knockdown cells were then cultured for 48 h, and the supernatant was collected. Three hundred cells were plated in each six-well plate. After being incubated with supernatant from different treaded THP-1 for 14 days, colony formation was observed and photographed.

### Enzyme-linked immunosorbent assay (ELISA)

THP-1 cells were transfected with FAM198B siRNA or control siRNA, combined with IL-4 and cultured for 72 hours, and the cell supernatant was collected. Cytokines were measured by ELISA. The levels of IL-10, TGF-β1, IL-12P70, IL-1β, and TNF-α and were measured using ELISA kits (Abcam, Cambridge, UK) following the manufacturer’s instructions. The 2-tailed Student’s t-test was used to determine statistical significance. A p-value less than 0.05 was considered to indicate a statistically significant difference between groups.

### Statistical analysis

All data and error bars are presented as the mean ± SD from at least three independent experiments (n = 3). Differences between two groups were evaluated by a two-tailed Student’s t-test. The indicated P values (*P < 0.05, **P < 0.01 and ***P < 0.001) were considered statistically significant.

## Results

### Increased macrophage expression of FAM198B in CRC is associated with poor prognosis

**Figure 1.**
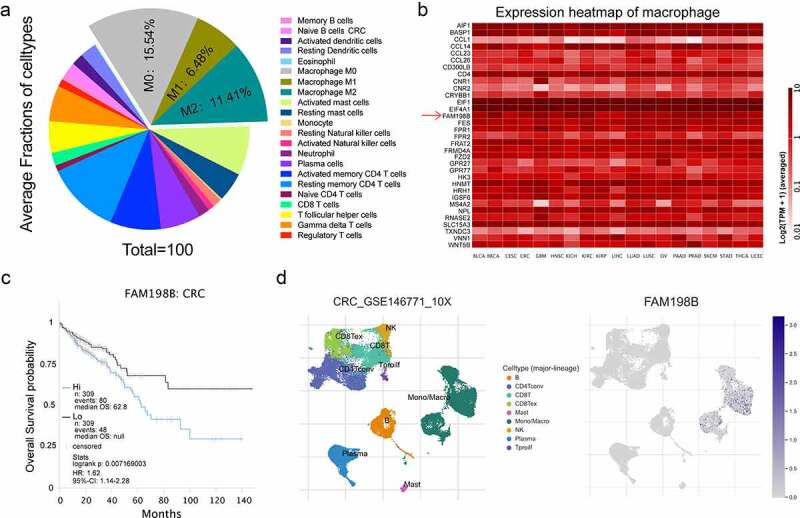
Increased FAM198B expression in colorectal cancer (CRC) macrophages is associated with poor prognosis. Average fractions of immune cell types in CRC (Figure 1(a)); Heatmap of macrophage gene expression in CRC (Figure 1(b)); Survival analysis of macrophage FAM198B expression in CRC (Figure 1(c)); FAM198B was highly expressed in macrophages in CRC (Figure 1(d)).

During cancer development, the changes in cells that infiltrate the tumor microenvironment can influence tumor progression [21,22]. To evaluate the distribution of immune cells in CRC, we first used cell-type fractions analysis through The Cancer Immunome Atlas Database (TCIA). Macrophages were found to be as high as 33.43% of whole immunocytes among the immune infiltration of CRC (Figure 1(a)). To further interrogate the importance of macrophages, we characterized the gene expression of macrophages using a heat map, and we found that the expression of many genes, including the FAM198B gene in macrophages, were upregulated in CRC and other cancers (Figure 1(b)). Furthermore, we found that only the expression of FAM198B in macrophages was negatively correlated with prognosis of CRC (Figure 1(c), Figure S1) (TCIA [23], https://tcia.at/home). From the TISCH database (TISCH [19], http://tisch.comp-genomics.org/home), FAM198B was also found to be highly expressed in macrophages in CRC (Figure 1(d)). These data indicate FAM198B may play an important role in TAMs in CRC.

### The function of FAM198B in macrophages

**Figure 2.**
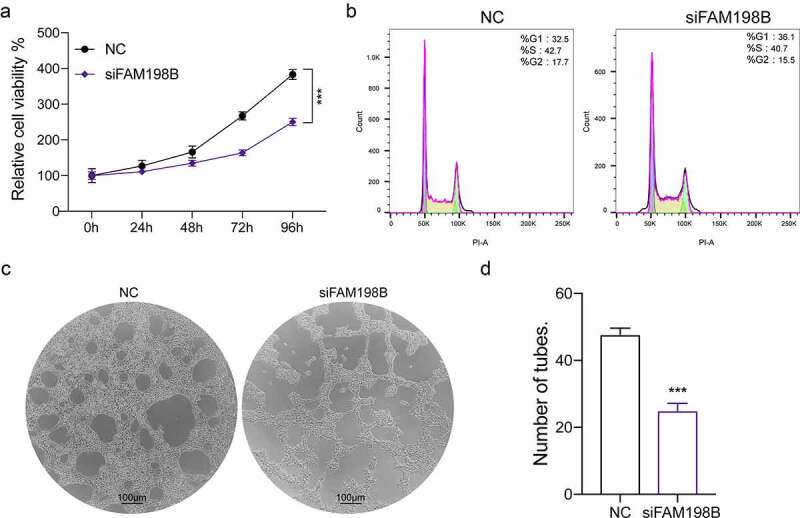
The function of FAM198B in macrophages. The human monocytic cell line, THP-1, was treated with FAM198B siRNA and proliferation, cell cycle, and angiogenesis were evaluated. Cell activity was reduced by FAM198B knockdown relative to a control siRNA knockdown (n = 3) (Figure 2(a)). Cell cycle distribution in THP-1 cells was assessed by flow cytometry after staining with propidium iodide (Figure 2(b)). *In vitro* angiogenesis: HUVECs were seeded in Matrigel-coated 24-well plates incubated with a supernatant harvested from differently treated THP-1 cells. The cells were allowed to culture for 6 h on Matrigel in conditional media. The effects of conditioned media on the capillary structures formed by HUVECs were analyzed, and the tube number was counted (n = 3) (Figure 2(c,d)).

To further investigate the role of FAM198B in macrophages, we treated THP-1 mono-macrophages with FAM198B siRNA and measured macrophage proliferation, cell cycle, and the ability to promote angiogenesis. FAM198B knockdown inhibited the proliferation of THP-1 cells, as determined by CCK-8 assay (Figure 2(a)). FAM198B knockdown also increased the proportion of THP-1 cells in G1 phase and decreased the proportion in S and G2 phases, indicating that the cell cycle was prolonged (Figure 2(b)). Similarly, the angiogenic ability of the THP-1 cells was also inhibited by FAM198B siRNA (Figure 2(c,d)).

### FAM198B promotes M2 macrophage polarization

**Figure 3.**
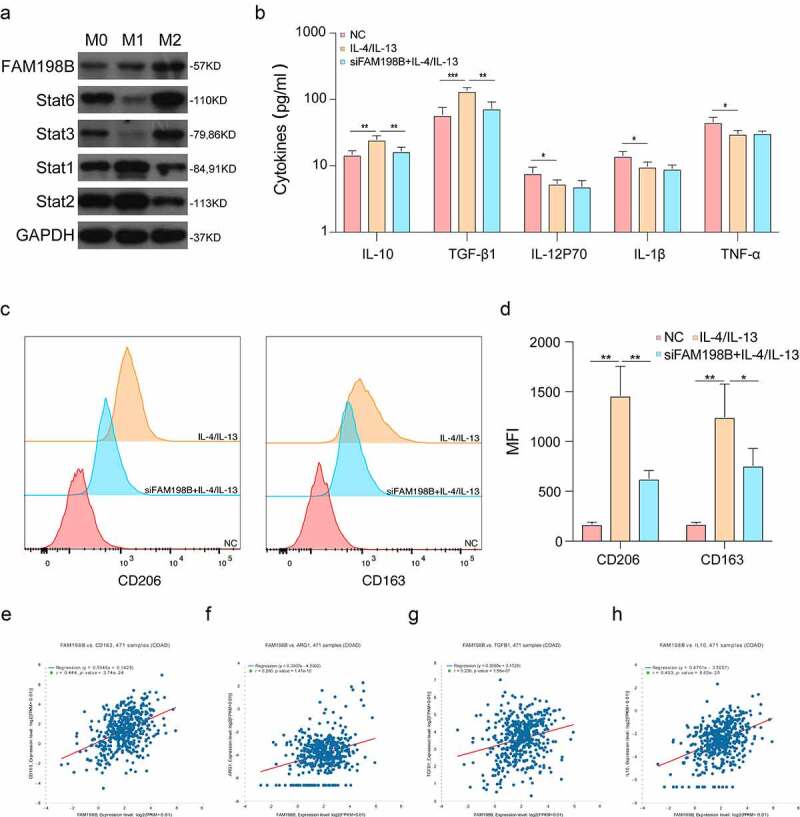
FAM198B promotes M2 macrophage polarization. THP-1 induced by different conditions: LPS/INF-γ-induced M1-type macrophages, and IL-4/IL-13-induced M2-type macrophages. Western blot detection of Stat1, Stat2, Stat3, Stat6, and FAM198B protein expression (Figure 3(a)). ELISA detection of expression of different cytokines (n = 3) (Figure 3(b)). The expression of surface receptors CD163 and CD206 of M2 macrophages was evaluated by Flow cytometry (n = 3) (Figure 3(c,d)). The correlation between FAM198b and M2-type marker genes (Figure 3(e-h)).

TAMs that promote angiogenesis and cancer progression are M2 macrophages; therefore, we evaluated whether FAM198B contributes to macrophage polarization. We evaluated the expression of FAM198B in M0-macrophages, M1-macrophages (LPS/INF-γ-induced), and M2-macrophages (IL-4/IL-13-induced) [24]. M2-like macrophages (IL-4/IL-13 incubated) showed an increase in expression of FAM198B (Figure 3(a)). To further illustrate the role of FAM198B in macrophage polarization, we examined whether M2-type polarization could be regulated by FAM198B. IL-4/IL-13 increased the secretion of M2-related cytokines (IL-10 and TGF-β1), and FAM198B knockdown by siRNA effectively blocked these changes. However, M1-related cytokines (IL-12P70, IL-1β and TNF-α) were not significantly changed (Figure 3(b)). The increased expressions of surface receptors CD163 and CD206 of M2 macrophages were also inhibited by FAM198B siRNA (Figure 3(c,d)), while M1-related surface receptors CD16 and CD32 were not significantly changed (Fig. S2 A, B). Further analysis by Starbase2.0 [25] (http://starbase.sysu.edu.cn) on the relation of FAM198B and M2-type marker genes showed that FAM198B is highly consistent with M2-type marker genes. We speculate that FAM198B is likely to regulate M2 polarization of TAMs (Figure 3(e-h)).

### High expression of FAM198B in macrophages promotes the progression of CRC in vitro

**Figure 4.**
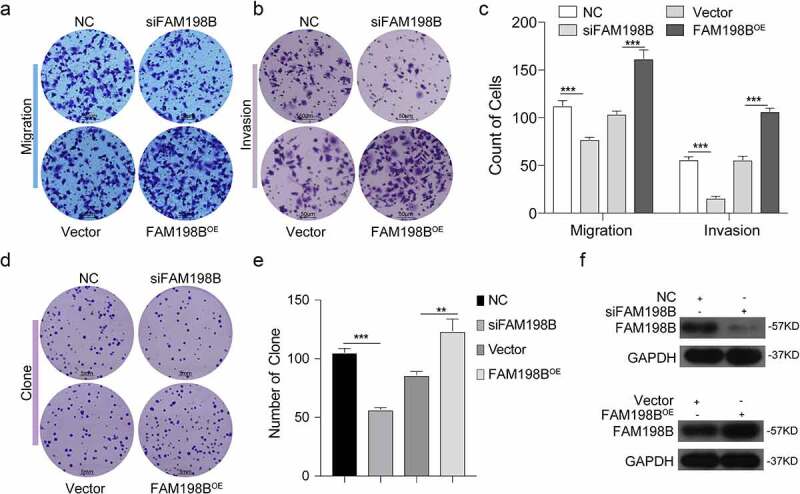
High expression of FAM198B in macrophages promotes the development of CRC. THP-1 cells were transfected with FAM198B siRNA or overexpression plasmid to knockdown or overexpress FAM198B, and were co-cultured with CRC cell lines for 72 h to detect the ability of macrophages to affect migration, invasion (n = 3) (Figure 4(a-c)), and cloning (n = 3) of CRC cells (Figure 4(d,e)). FAM198B transfection efficiency (Figure 4(f)).

We evaluated the role of FAM198B in inducing M2 polarization on CRC progression *in vitro*. We performed co-culture of macrophages with CRC cells for 72 hours to detect the effects of macrophages on CRC migration (Figure 4(a)), invasion (Figure 4(b,c)), and clonogenic ability (Figure 4(d,e)). Western blot was used to determine the transfection efficiency of THP-1 cells with FAM198B siRNA or FAM198B overexpression plasmid (Figure 4(f)). The data show that the high expression of FAM198B in macrophages increases the migration, invasion, and colony formation of CRC cells, while the knockdown of FAM198B in macrophages inhibits macrophage-induced increases in migration, invasion, and colony formation.

### Protein–protein interaction enrichment analysis of FAM198B

The proteins that interact with FAM198B were retrieved through THE Pathway, and the signal pathway enrichment analysis of these proteins was performed using IPP (protein–protein interaction, carried out with the following databases: STRING [26], BioGrid [27], OmniPath [28], and InWeb_IM [29]) (Fig. S3 A).

### Identification of genes that may be regulated by FAM198B in macrophage polarization

We further evaluated how FAM198B regulates macrophage polarization. We found that the M2-PID SMAD2/3 NUCLEAR PATHWAY had the greatest correlation with FAM198B (Fig. S3 A). Further analysis by Starbase2.0 [25] (http://starbase.sysu.edu.cn) on the relationship between SAMD2, FAM198B, and other macrophage M2 marker genes showed that FAM198B is highly consistent with these genes. We speculate that FAM198B is likely to regulate M2 polarization of macrophages by targeting SMAD2 (Fig. S3 B, C, D).

### FAM198B regulates the M2 polarization of macrophages by targeting SMAD2

**Figure 5.**
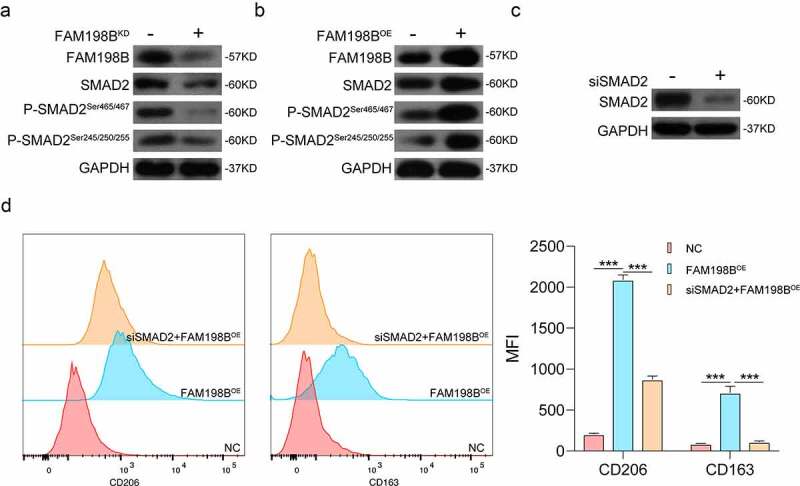
FAM198B regulates the M2 polarization of macrophages by targeting SMAD2. After overexpression or knockdown of FAM198B, western blot was used to evaluate the expression levels of FAM198B, SMAD2, and P-SMAD2. The expression levels of SMAD2 and P-SMAD2 in the FAM198B overexpression group were higher than that in the knockdown group, with a trend of synchronization (Figure 5(a,b)). After knocking down SMAD2 by siRNA, the MFI of CD206 and CD163 in the SMAD2 knockdown group was lower than in the FAM198B overexpression group (n = 3) (Figure 5(c,d)).

We investigated whether SMAD2 expression is regulated by FAM198B. After overexpression or knockdown of FAM198B in THP-1, western blot was used to evaluate the expression levels of FAM198B, SMAD2, and P-SMAD2. The protein level of SMAD2 and P-SMAD2 in macrophages were significantly increased in the FAM198B overexpression group and decreased in cells with FAM198B knockdown (Figure 5(a)). More importantly, the FAM198B-mediated increases in CD206 and CD163 levels were significantly suppressed by simultaneous SMAD2 silencing, which further supports our hypothesis that FAM198B induces M2 polarization of macrophages via the SMAD2 pathway (Figure 5(b-d)).

## Discussion

In this study, we have shown that FAM198B activates macrophages to an M2-like phenotype via the SMAD2 pathway, which leads to CRC cell proliferation, migration, and invasion. This is the first study to demonstrate a mechanistic role for FAM198B in mediating the pro-tumor activities of TAMs in colorectal cancer.

Through analyzing the data from TCIA, we found FAM198B is highly expressed in macrophages (Figure 1(b)). More specifically, the increased macrophage FAM198B expression in colorectal cancer (CRC) is associated with poor prognosis (Figure 1(c)). However, previous research described FAM198B as a prognostic marker for LUAD, demonstrating that DAM198B inhibited LUAD metastasis by blocking ERK-mediated MMP-1 expression [10]. Inhibition of FAM198B expression promotes the proliferation and migration of ovarian cancer cells, and FAM198B also acts as a tumor suppressor gene and is down-regulated in ovarian cancer [9]. These contradictory reports suggest that FAM198B may play dual roles, *i.e*. as an oncogene role or as a tumor suppressor gene in different cancer contexts, possibly also indicating that FAM198B may have different effects in different cells. Although knowledge of its role in cancer is limited, the evidence suggests that FAM198B plays an important role, either as a tumor suppressor or as an oncogene. In macrophages, FAM198B knockdown leads to a profound decrease of angiogenesis, reduces cell viability, causes cell cycle arrest, and abrogates the induction of M2-type macrophages by IL-4/IL-13 (Figures 2 and 3).

The tumor microenvironment is important in tumorigenesis and cancer development [30,31]. Macrophages have complex and diverse tumor-promoting roles within the tumor microenvironment, and TAMs are a potential therapeutic target [32]. Co-culture of FAM198B-overexpressing macrophages with cancer cells resulted in a further increase of CRC migration and invasion (Figure 4). M2-macrophages are thought to facilitate tumor progression, while M1-macrophages promote a strong immune response and eliminate cancer cells. M2-polarized TAMs promote cancer progression via the secretion of pro-tumor cytokines and the subsequent production of tumor growth factors [3,33].

The role of FAM198B as a tumor-associated gene has been studied in multiple types of cancers, including lung cancer and ovarian cancer [9,10]. However, the functional role of FAM198B in colorectal cancer had not yet been identified. Here, we performed pathway analysis and evaluated associations of FAM198B with particular genes (Fig. S 3, 4). FAM198B may act to favor tumor progression by interacting with numerous genes, such as the p-ERK/MMP-1 signaling pathway in lung adenocarcinoma [9], and S100A3, PCDH9, and SEMA3A genes in ovarian cancer [8,34]. A rescue experiment was performed to investigate the effects of FAM198B in the SMAD2 pathway. Importantly, we revealed that, in macrophages transfected with SMAD2 siRNA, the effects of FAM198B overexpression on macrophage-promoted cancer cell migration and invasion were impeded. The SMAD2 rescue experiments proved that FAM198B regulates M2-like polarization of TAMs mainly by targeting SMAD2 (Figure 5). These data clarify the relationship between TAMs and cancer cells, whereby FAM198B acts through the SMAD2 pathway to drive TAMs-mediated cancer cell migration. Data from several studies suggest that the expression of constitutively active SMAD2/3 significantly improves the efficiency of reprogramming conversions in macrophages [35]. Macrophages promote endothelial-to-mesenchymal transition via MT1-MMP/**TGF-β1** after myocardial infarction, and CD51+ macrophages promote cancer stem cell properties through the TGF-β1/Smad2/3 axis in pancreatic cancer [36,37]. This study suggests that FAM198B promotes macrophage polarizing to M2-type via a SMAD2-mediated mechanism, whereby M2-type macrophages secrete significantly higher levels of cytokines such as IL-10,TGF-β1 to neighboring CRC cells. TGF-β1 is a major regulator of survival, proliferation, and metastasis in cancer cells [38] and is a driver of TAMs-mediated cancer cell migration. These results may provide a mechanistic explanation for the clinical association between FAM198B expression in macrophages in colorectal cancer and higher potential for invasion and metastasis.

We further found that colorectal cancer patients with low expression of FAM198B in TAMs had a better prognosis than those with high FAM198B expression in TAMs, suggesting that FAM198B may serve as a potential biomarker for prognostic assessment. These data suggest that FAM198B/SMAD2 modulates M2-like polarization of TAMs to regulate the secretion of cytokines that affects numerous aspects of colorectal cancer cell biology. Additional studies are needed to determine how SMAD2 is transcriptionally and phosphorylation regulated in CRC-associated macrophages. We will further analyze the role of FAM198B/SMAD2 in colorectal cancer by incorporating more clinical samples in further studies.

## Conclusion

Our data reveal a vital role for FAM198B in TAMs in CRC. High expression of FAM198B in TAMs of colorectal cancer was associated with poor overall survival, suggesting that FAM198B in TAMs may be an effective diagnostic and prognostic marker for CRC.

## Supplementary Material

Supplemental Material

## Data Availability

The data that support the findings of this study are available from the corresponding authors upon reasonable request.
