# Supplementary material for: FAM198B promotes colorectal cancer progression by regulating the polarization of tumor-associated macrophages via the SMAD2 signaling pathway
Source: Bioengineered. 2022 May 19;13(5):12435–45. doi: 10.1080/21655979.2022.2075300 (PMC9276016; doi:10.1080/21655979.2022.2075300)
Supplement: Supplemental Material [file KBIE_A_2075300_SM2875.zip › Language certificate.pdf]

www.elixigen.com

4/21/2022

Editorial Certification

This document certifies that the manuscript titled "FAM198B promotes colorectal cancer progression by regulating the polarization of tumor-associated macrophages via the SMAD2 signaling pathway" was edited for proper English language, grammar, punctuation, spelling, and overall style by one or more of the highly qualified native English speaking editors at ELIXIGEN.

Neither the research content nor the authors' intentions were altered in any way during the editing process. Documents receiving this certification should be English-ready for publication - however, the author has the ability to accept or reject our suggestions and changes. To verify the final ELIXIGEN edited version, please contact ELIXIGEN at support@elixigen.com.

*We are NOT responsible for any errors in the added content to our revised version after this date.
