# Supplementary material for: FAM198B promotes colorectal cancer progression by regulating the polarization of tumor-associated macrophages via the SMAD2 signaling pathway
Source: Bioengineered. 2022 May 19;13(5):12435–45. doi: 10.1080/21655979.2022.2075300 (PMC9276016; doi:10.1080/21655979.2022.2075300)
Supplement: Supplemental Material [file KBIE_A_2075300_SM2875.zip › Supplementary Figure and legendsclean.docx]

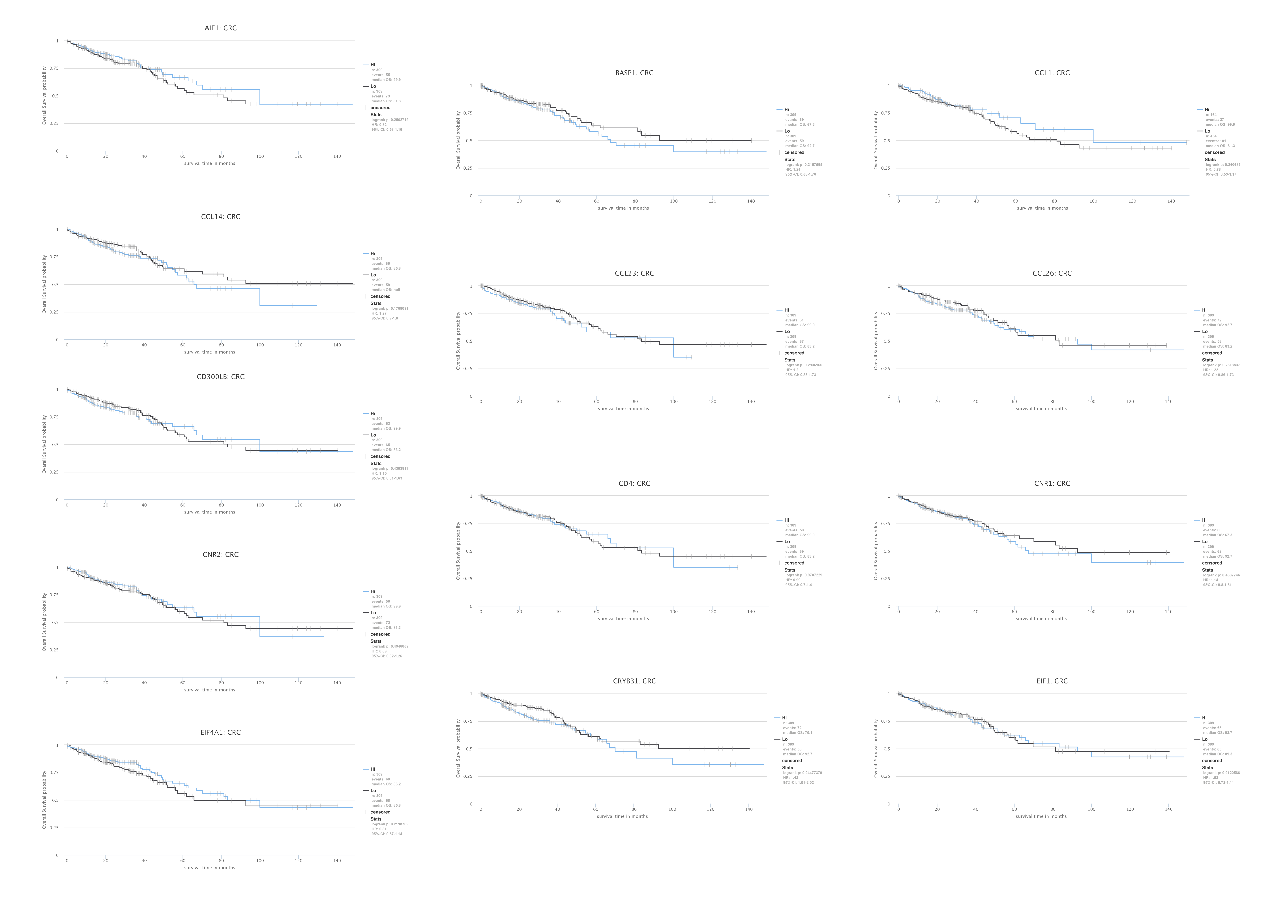


**Supplementary Figure 1 The expression of ralated gene in macrophages and prognostic relationship in CRC**


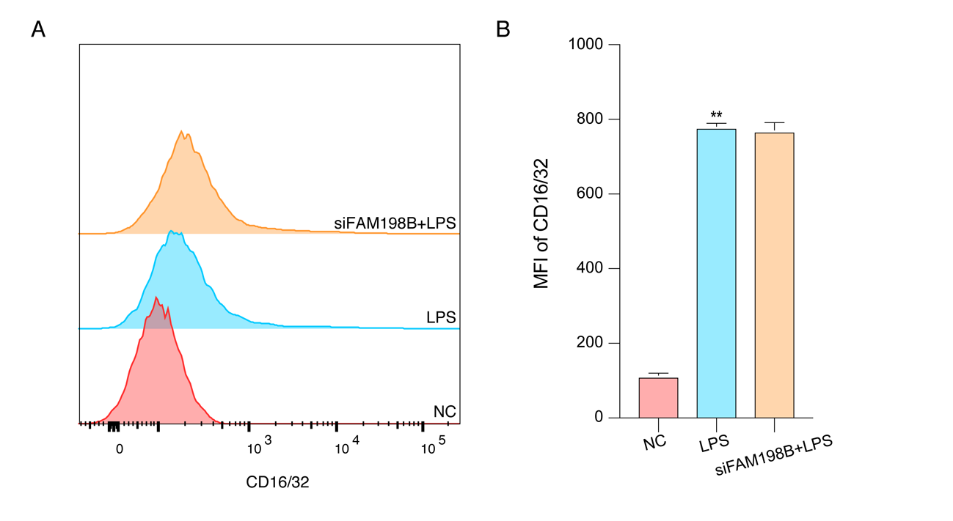


**Supplementary Figure 2 The surface receptors CD16 and CD32 test by Flow cytometer.**


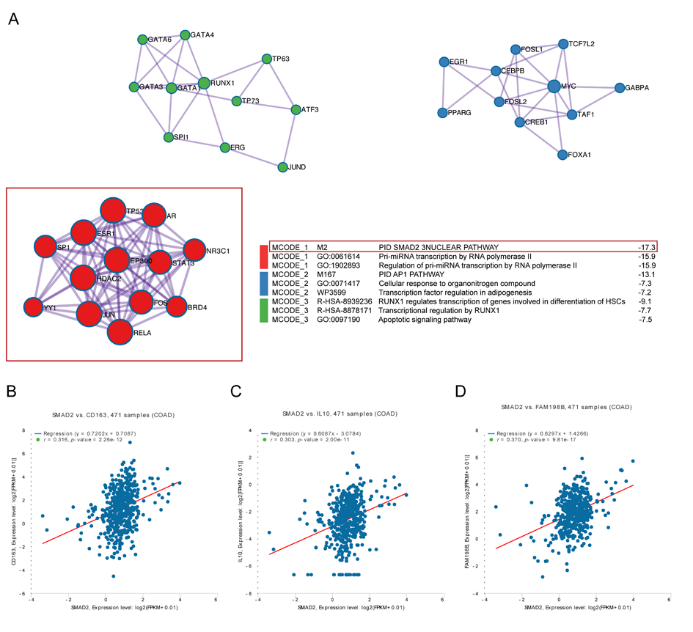


**Supplementary Figure 3 Genes that may be regulated by FAM198B in macrophage polarization.**

M2 (PID SMAD2/3 NUCLEAR PATHWAY) has the greatest correlation (Fig. S2 A). The relation of SAMD2, FAM198b, and the other macrophage M2 Marker genes showed that FAM198B is highly consistent with these genes (Fig. S2 B, C, D).


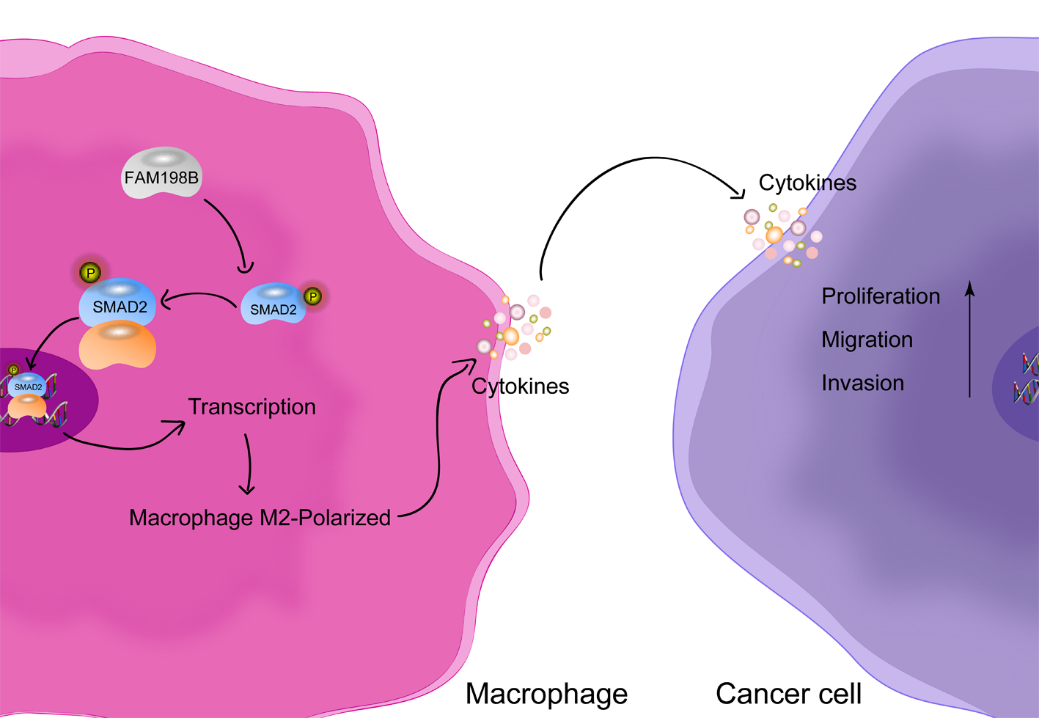


**Supplementary Figure 4 The molecular mechanisms for the function of FAM198B in TAMs of CRC**
